# Supplementary material for: A whole-animal phenotypic drug screen identifies suppressors of atherogenic lipoproteins
Source: eLife. 2026 Aug 27;14:RP105314. doi: 10.7554/eLife.105314 (PMC13521566; doi:10.7554/eLife.105314)
Supplement: Supplementary file 2 [file elife-105314-code1.zip › JHDL_primary_result/POMIFERIN/POMIFERIN_JHDL_4_005_005_2019_05_10_11_07.pdf]

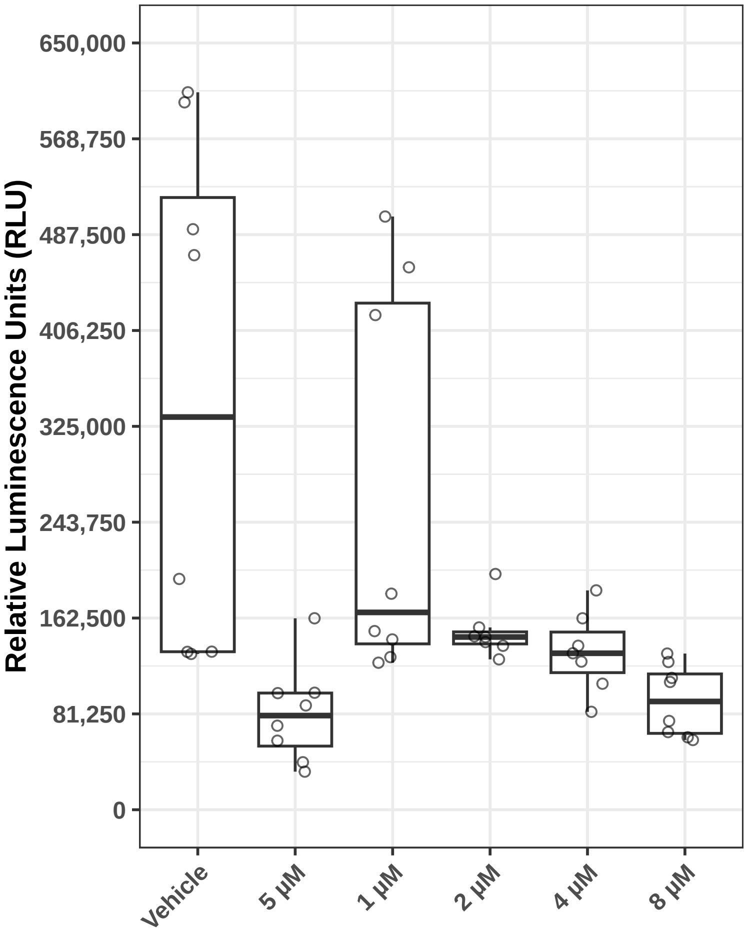

650,000
568,750
487,500
406,250
325,000
243,750
162,500
81,250
0
Relative Luminescence Units (RLU)
Vehicle
5 µM
1 µM
2 µM
4 µM
8 µM
